# Mental health of working parents during the COVID-19 pandemic: can resilience buffer the impact of psychosocial work stress on depressive symptoms?

**DOI:** 10.1186/s12889-022-14582-y

**Published:** 2022-12-26

**Authors:** Stephanie Brym, Judith T. Mack, Victoria Weise, Marie Kopp, Susann Steudte-Schmiedgen, Susan Garthus-Niegel

**Affiliations:** 1 Institute and Policlinic of Occupational and Social Medicine, Faculty of Medicine, Technische Universität Dresden, Dresden, Germany; 2 Department of Psychotherapy and Psychosomatic Medicine, Faculty of Medicine, Technische Universität Dresden, Fetscherstr. 74, 01307 Dresden, Germany; 3 Institute for Systems Medicine and Faculty of Human Medicine, MSH Medical School Hamburg, Hamburg, Germany; 4 Department of Childhood and Families, Norwegian Institute of Public Health, Oslo, Norway

**Keywords:** Working parents, Work-privacy conflict, Effort-reward imbalance, Resilience, Depressive symptoms, Moderation, DREAM study

## Abstract

**Background:**

The COVID-19 pandemic has confronted working parents with an accumulation of stressors regarding changes in work, family, and social life, putting their mental health at risk. Stressors include altered working conditions such as working from home or changes in working hours as well as the difficulty to reconcile work and childcare due to the closure of childcare facilities. The present study examined the relationship of psychosocial work stress (i.e., work-privacy conflict and effort-reward imbalance at work) and depressive symptoms in working parents and whether this association was moderated by individual resilience.

**Methods:**

Data of the present study (*n* = 452) were collected in Germany between May and June 2020 as part of the DREAM_CORONA_ study. A subsample of working mothers (*n* = 191) and fathers (*n* = 261) completed the subscale for work-privacy conflict (WPC) of the Copenhagen Psychosocial Questionnaire, the Effort-Reward Imbalance (ERI) Questionnaire, the Connor-Davidson Resilience Scale (CD-RISC), and the Edinburgh Postnatal Depression Scale (EPDS). Multiple linear regression analyses including moderation were performed, controlling for gender, working hours per week, and a lifetime history of depression as potential confounders.

**Results:**

Both WPC (β = 0.336, *p* < .001) and ERI (β = 0.254, *p* < .001) were significantly associated with depressive symptoms. Resilience moderated the relationship between ERI and depressive symptoms (β = − 0.101, *p* = .018), indicating that higher resilience weakened the relationship. However, this effect was not found regarding the relationship between WPC and depressive symptoms (β = 0.055, *p* = .167).

**Conclusions:**

The results highlight the need for measures to reduce psychosocial work stressors such as WPC and ERI during the COVID-19 pandemic on the one hand and to promote resilience on the other hand. The findings partially support the potential protective role of resilience buffering the association between psychosocial stress and mental health in working parents. Longitudinal studies are needed to confirm this effect.

**Supplementary Information:**

The online version contains supplementary material available at 10.1186/s12889-022-14582-y.

## Background

In March 2020, the World Health Organization (WHO) declared the outbreak of the COVID-19 virus a pandemic [1]. With the aim to control the virus, Germany among other countries imposed strict political measures to contain infections. As a result, individuals were faced with various challenges and major changes regarding family life, work, and their social interaction. Emerging stressors due to altered working conditions included being forced to work from home, the need to adapt to changes in working hours (e.g., working overtime or working short-time), financial strains, and insecurities regarding the future of one’s employment [2–4]. Additionally, four million working parents were affected by the abrupt closure of childcare facilities in Germany [5]. Schools and day-care facilities were closed nationwide from March 2020 to May 2020, only offering emergency childcare. Even after the strict closures were lifted, childcare services were not available to all families again [6]. As a consequence, especially working parents were confronted with fundamental disruptions of their normal life both at work and at home. While adapting to changes in work demands, reconciling childcare and family responsibilities with work became more difficult [7]. This accumulation of psychosocial work stressors may put the mental health of working parents at risk.

**Fig. 1:**
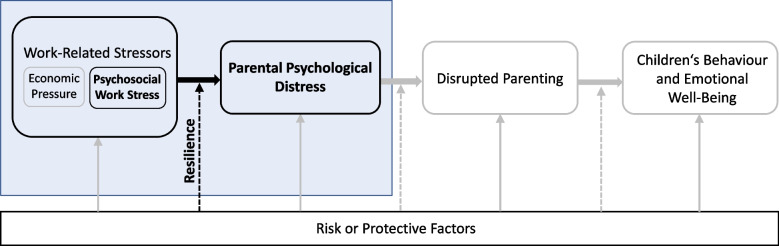
Applying Family Stress Models to the situation of working parents during the COVID-19 pandemic. *Note.* Model adapted from Conger & Conger [29], Conger [23], and Masarik & Conger [24]

Studies shortly initiated after the outbreak of the pandemic already indicated a negative impact on mental health in the general population. Results revealed a significant deterioration of mental well-being, an increase of depressive symptoms and anxiety symptoms associated with the outbreak, as well as highly elevated stress levels [8–10]. This impact did not spare parents as first studies have shown: Parents reported high levels of psychological distress [11, 12], a deterioration of their mental health [13, 14], and an elevation in maternal depression and anxiety compared to results prior to the pandemic [15]. Especially the well-being of mothers and families with younger children seemed to be impaired [13, 16, 17]. Taking a closer look at the group of working parents during the COVID-19 pandemic, changes in working conditions have been found to be associated with lower well-being [14] and exhaustion was particularly elevated in mothers of pre-school aged children [18]. At the same time, protective factors regarding parents’ mental health such as high individual resilience have been shown to be negatively associated with outcomes such as parenting-related exhaustion or depression [12, 19]. Considering the circumstances working parents are confronted with during the COVID-19 pandemic, it is essential to take a closer look at specific potential risk and protective factors regarding their mental health. Examining parents’ mental health is not only of great importance since it affects mothers and fathers as individuals but also, because it may affect mental health within their family system. Poor mental health of parents is associated with adverse child outcomes like both internalizing and externalizing problems as well as general psychopathology [20, 21]. Indication of such negative effects of parental distress on children’s health, behaviour, and emotion regulation have already been suggested during the pandemic [11, 22]. Models such as the Family Stress Model [23, 24] and a process model suggested by Belsky [25] point out the crucial role of parents’ well-being regarding family mental health, offering a theoretical framework to integrate both potential stressors and protective factors, the well-being of parents and children, as well as their interrelations. The models describe cascading effects of stressors like economic hardship or contextual stress (e.g., work stress), eliciting parental psychological distress. Parental distress in turn may first affect parents’ individual mental health, amounting to negative effects on parenting and child behaviour and well-being [25, 26]. Especially parental depression is considered a risk factor for adverse child outcomes [27, 28]. However, protective factors can buffer the family stress process [24]. Applying family health models to the context of the COVID-19 pandemic, psychosocial work stress due to fundamental changes in work and family life might represent a potential stressor for working parents, threatening their mental health, and thereby risking triggering cascading effects (Fig. 1).

Despite the findings that particularly working parents are at high risk for mental distress during the ongoing pandemic, previous studies considered work only in a very broad manner, lacking the assessment of psychosocial work stressors in more detail and neglecting the potential protective role of resilience. The current study therefore aims at closing this gap to strive for a better understanding of mental health of working parents and to identify possible protective factors in the context of a pandemic.

### Psychosocial work stress

Psychosocial work stress can stem from a variety of sources (e.g., job content, lack of autonomy, work intensity, working hours, work-life imbalance) [30]. Considering the multitude of potential psychosocial stressors working parents were confronted with during the pandemic, two well-established concepts to explain the development of psychosocial work stress will be used: work-privacy conflict (WPC) and effort-reward imbalance (ERI).

#### Work-privacy conflict

Based on the spill-over theory [31], WPC, sometimes also referred to as work-family conflict, picks up on the negative interference of experiences at work with experiences in the private/ family domain [32, 33]. In the context of the COVID-19 pandemic, changes such as working from home threatened distinct boundaries between work and private life, risking an increase in conflicts between these two domains. There is well-established evidence for associations of WPC with poor well-being, burnout, and depression [33–36]. A first study conducted during the pandemic indicated an association of WPC with exhaustion among employees [18]. This highlights the urge to further examine the relationship of WPC with other indicators of poor mental health, such as depressive symptoms, particularly in working parents.

#### Effort-reward imbalance

The Effort-Reward Imbalance Model suggests that stress occurs if there is a lack of social reciprocity between effort and reward at the workplace, caused by an imbalance between the effort spent and the perceived reward received [37]. As a consequence, high effort and low reward may result in stress, which in turn increases the risk for various adverse health effects [37]. Type of rewards include salary or wage, career promotion or job security, esteem or recognition [38]. Considering the economic challenges due to the COVID-19 pandemic, receiving a reward at work that is perceived as appropriate can be severely threatened: Salaries might be reduced due to short-time work, career promotion may be scarce, and job security low, thereby potentially disturbing the balance of effort and reward at work. Research has shown that ERI is associated with psychological distress. Meta-analyses suggest a 1.5-fold increased risk of depressive disorders [39] and higher incidence of stress-related disorders among employees being subject to ERI [40]. During the pandemic, ERI has only been examined in individuals working in the health care sector, showing positive associations with depressive symptoms [41, 42].

### Resilience

The COVID-19 pandemic imposed an immense stress load on parents. However, some mothers and fathers seem to be more affected by such an adverse life event than others. While some individuals develop psychological symptoms, others seem to be able to deal with additional or unpredicted stressors and adapt to changes without showing mental problems. An underlying factor trying to explain why some people seem to be “immune” to stress is individual psychological resilience [43, 44]. Although there is no universally accepted definition, in summary, resilience can be understood as the capacity to adapt to adverse environmental conditions, the ability to show adaptive behaviour in response to numerous stress factors, and to maintain or regain mental health despite significant adverse stressors [45, 46]. Previous findings did not only reveal a negative association of high resilience with symptoms of depression and anxiety [47], but also emphasized its buffering effect on the impact of stress on depressive symptoms [48, 49]. Recent studies conducted during the COVID-19 pandemic support the protective role of resilience reporting inverse correlations with symptoms of depression as well as effects buffering the impact of stress [19, 50–52]. However, so far, little is known about the effect of resilience in working parents during the ongoing crisis and its influence on the association between psychosocial work stress and depressive symptoms.

### Aims and objectives

To close these gaps, this study will (1) examine the association between psychosocial work stress (ERI and WPC) and depressive symptoms in working parents during the COVID-19 pandemic and (2) investigate the potential moderating effect of resilience upon the association between psychosocial work stress and depressive symptoms. This appears to be especially important if we not only consider the well-being of parents themselves but also expand our view to the negative impact of parental distress on children’s well-being.

## Methods

### Participants and procedure

The present sample is part of the DREAM_CORONA_ study, an online sub-study of the large multi-method cohort Dresden Study on Parenting, Work, and Mental Health (**DREAM**; **DR**esdner Studie zu **E**lternschaft, **A**rbeit und **M**entaler Gesundheit). The DREAM study investigates the interplay of parental roles, work participation, and health-related outcomes in (expectant) mothers and fathers [53]. In response to the outbreak of the pandemic, the DREAM_CORONA_ study was established as an addition to the regular assessments. The DREAM_CORONA_ sub-study investigates experiences of (expectant) parents during the COVID-19 pandemic (e.g., isolation, school, and daycare closures, working from home) and its impact on family health, role distributions, and relationships. Only participants of the general cohort of the DREAM study who completed follow-up questionnaires via an online version were eligible (*n* = 1885). Pandemic restrictions hindered sending out study material and thereby reaching participants using the paper-pencil version. Due to feasibility reasons, parents of twins or multiples did not receive an invitation. Invitations for the online survey were sent out via mail on May 12, 2020, followed by two reminders after three and 6 days, respectively. The survey was open to response until October 1, 2020, and included questions regarding socio-demographic data, COVID-19-specific questions, and several questionnaires regarding social, work, and health factors. Of all invited parents, *n* = 1057 gave consent to participate. To control for changes in political restrictions, participants who responded to the survey after June 5, 2020, were excluded for the current analyses as with June 6, 2020, new COVID-19 regulations came into effect. Further exclusion criteria were not having children yet at the time of responding to the survey and no current employment. The following responses were accepted as being currently employed: working full-time, part-time, or in marginal employment, being in an irregular employment, in an apprenticeship, or doing a voluntary service. Being on employment ban was excluded as well as contradictory information such as working full-time and being on parental leave at the same time. In the last step, participants with incomplete data regarding either the beforementioned exclusion criteria or the outcome, i.e., depressive symptoms, were excluded (for flowchart see Additional file 1). Demographic information such as country of birth, education, and suffering from a lifetime history of depression were derived from data of the respective participant collected in previous assessments of the DREAM study.

### Instruments

#### Psychosocial work stress

Psychosocial work stress was operationalized by assessing WPC and ERI. Participants were asked to specifically refer to their current working conditions since February 2020, i.e., the time when the first effects of the impending pandemic emerged in this region. WPC was assessed through the corresponding scale of the Copenhagen Psychosocial Questionnaire [54, 55]. The scale contains seven items, which are rated on a 5-point Likert scale ranging from 1 (*to a very low degree*) to 5 (*to a very large degree*). Scores were transformed to fit the range 0–100, with higher scores indicating a stronger interference of work with private life. With reference to Nübling and colleagues [55], missing values were replaced by the individual’s mean if the participant responded to at least 50% of the items. The scale showed good internal consistency (Cronbach’s *α* = .86).

The Effort-Reward-Imbalance Questionnaire [56, 57] was used to assess ERI at work. The questionnaire consists of three subscales: *effort*, *reward*, and *overcommitment*. Regarding the present research question, we used a short version of the questionnaire [57], including the *effort* (three items) and *reward* (seven item) scales. All items were rated on a 4-point Likert scale with responses ranging from 1 (*totally agree*) to 4 (*totally disagree*). Sum scores for each scale were calculated, with higher scores reflecting higher effort and higher reward, respectively. Stress was operationalized by the relationship between effort and reward, the ERI ratio. To compute the ERI ratio, the *effort* sum score was divided by the *reward* sum score, multiplied by a factor to correct for the unequal number of items of the two scales [58]. A ratio below 1 indicates that the reward exceeds the effort while a ratio equal to 1 indicates a balance of effort and reward. Values greater than 1 indicate an imbalance of high effort and low reward which is considered stressful [59]. To improve statistical power, we used the ERI ratio as a continuous variable in regression analyses, calculating the logarithm of the ERI ratio [59]. Compared to the raw ratio, the log-transformed ratio places the inverse imbalance of the same magnitude in the same distance from 1 [58, 60] and assigns a meaningful value to zero which is helpful for interpreting main effects in a moderated regression analysis. Any missing values regarding the effort scale led to exclusion of the participant for the analyses. Regarding the reward scale, missing values were replaced by the individual’s mean if the participant replied to at least four of seven items. Both scales showed sufficient internal consistencies (Cronbach’s *α*_effort_ = .66, Cronbach’s *α*_reward_ = .77).

#### Resilience

The Connor-Davidson Resilience Scale (CD-RISC) [61] was used to assess individual resilience as the ability to cope with internal and external stressors. We used the short version consisting of ten items. Response categories range from 1 (*not true at all*) to 5 (*true nearly all of the time*), with higher scores indicating greater resilience. Missing values were replaced by the individual’s mean if at least 80% of the items were answered. Participants were asked to refer their responses to the previous month. Internal consistency was good (Cronbach’s *α* = .86).

#### Depressive symptoms

Depressive symptoms were assessed using the Edinburgh Postnatal Depression Scale (EPDS) [62, 63]. Even though the questionnaire was designed to assess depressive symptoms in women during the perinatal period, it has been shown to be valid for the assessment in non-postnatal women and fathers as well [64–66]. The scale contains ten items with four response categories assessing the frequency of the described symptoms during the previous 7 days. The sum score of all items was calculated with higher scores of the EPDS indicating stronger depressive symptoms. For the sample description, the most common cut-off scores were used to distinguish between indication of the absence or low symptoms, respectively, (≤ 9), minor depression [10–12], and major depression (≥ 13) [62, 63]. Missing values were replaced by the individual’s mean value to calculate the sum score if at least 80% of the items were answered. Reliability was good (Cronbach’s *α* = .86).

All measures rely on self-report. For all questionnaires, the corresponding validated German version was used.

### Confounders

Based on prior evidence and significant correlations with the outcome, i.e., depressive symptoms, potential confounders were selected. Following these criteria, gender, number of working hours per week, and lifetime history of depression (LHD) were included as potential confounders in all regression analyses. LHD was assessed as a dichotomous variable, indicating whether participants had a LHD or not. The variable is based on participants’ data from the general DREAM study.

### Data analysis

First, scores for all scales were calculated and checked for outliers. Second, descriptive analyses of sociodemographic variables, selected confounders (gender, number of working hours per week, LHD), the predictors (WPC, ERI), moderator (resilience), and outcome (depressive symptoms) were carried out. To test for gender differences, t-tests for independent samples were computed for all. Third, Pearson correlational analyses were conducted to explore the associations of all included variables. Fourth, multiple linear regression models using forced entry were conducted to investigate the association between psychosocial work stress and depressive symptoms and the moderating role of resilience while controlling for the selected confounders. To make coefficients more interpretable, the values of the respective predictor and moderator were centered around their mean prior to analysis. WPC and ERI were tested in separate models to individually determine their association with the outcome and their interaction with resilience. The procedure of entering the variables in the regression models was identical for both measures of psychosocial work stress. In the first model, only potential confounders were included to examine their influence on the outcome. In the next step, the respective indicator of psychosocial work stress was added, followed by resilience and the interaction term (psychosocial work stress x resilience) to examine moderation. Standard errors and 95% confidence intervals (CI) of B were based on bootstrapping with 1000 iterations. CI were bias corrected and accelerated. The level of significance was set to *p* < .05.

Before the final regression analysis, data were examined regarding extreme values and the main assumptions for linear regression according to Field [68] were checked. All analyses were carried out using IBM SPSS Statistics (Version 27.0). Due to missing data, *n* varied slightly between the different analyses.

## Results

### Sample characteristics

The final sample consisted of *n* = 452 parents (42.3% mothers, 57.7% fathers; Table 1). Mean age was *M* = 33.90 years (*SD* = 4.69) and participants were between 21 and 55 years old. Most participants were born in Germany (96.7%), in a permanent relationship (98.2%), and had one child (78.8%). With 68.6% of all participants holding a university degree, the sample consisted of women and men with a higher educational level compared to the average population of Dresden [69]. Regarding current employment, 60.6% indicated to work full-time, and 36.5% were working part-time. The distribution of employment status between genders was not equal, as 82.4% (16.1%) of fathers were working full-time (part-time) compared to 30.9% (64.4%) of mothers, respectively. However, this distribution is representative for the general population in Germany [70]. Mean working hours per week were *M* = 35.77 (*SD* = 10.85), with mothers working significantly less hours per week than fathers (*M*_mothers_ = 31.50, *M*_fathers_ = 38.89; *t*(450) = − 6.392, *p* < .001). Regarding changes due to the outbreak of the pandemic, 10.6% of the parents reported to be affected by working short-time, and 62.8% by working from home.

**Table 1:** Sample description

Sample characteristics	Mothers (*n* = 191)	Fathers (*n* = 261)	Total sample (*n* = 452)
**Age**	32.98 ± 4.06 (21–43)	34.56 ± 5.00 (24–55)	33.90 ± 4.69 (21–55)
**Country of birth**			
Germany	183 (96.3)	254 (98.1)	437 (97.3)
Other	7 (3.7)	5 (1.9)	12 (2.7)
**Permanent relationship**			
Yes	185 (96.9)	259 (99.2)	444 (98.2)
No	6 (3.1)	2 (0.8)	8 (1.8)
**Children**			
One	150 (78.5)	206 (78.9)	356 (78.8)
Two	39 (20.5)	45 (17.3)	84 (18.5)
Three or more	2 (1.0)	10 (3.8)	12 (2.7)
**Educational level**			
No university degree	56 (29.3)	81 (31.6)	137 (30.6)
University degree	135 (70.7)	175 (68.4)	310 (69.4)
**Employment status**^a^			
Full-time employment	59 (30.9)	215 (82.4)	274 (60.6)
Part-time employment	123 (64.4)	42 (16.1)	165 (36.5)
Marginal employment	4 (2.1)	5 (1.9)	9 (2.0)
Irregular employment	5 (2.6)	4 (1.5)	9 (2.0)
Apprenticeship	5 (2.6)	2 (0.8)	7 (1.5)
Voluntary service	1 (0.5)	–	1 (0.2)
**Working conditions**			
Working hrs/w	28.27 ± 11.29 (2–60)	35.48 ± 12.33 (2–72)	32.44 ± 12.42 (2–72)
Short-time work (yes)	16 (8.4)	32 (12.3)	48 (10.6)
Home office (yes)	121 (63.4)	163 (62.5)	284 (62.8)
**LHD**			
Yes	26 (13.8)	31 (11.9)	57 (12.7)
No	162 (86.2)	229 (88.1)	391 (87.3)
**WPC**^b^ (0–100)	40.11 ± 22.69 (0–100)	36.71 ± 20.96 (0–100)	38.15 ± 21.75 (0–100)
**ERI**			
Effort scale (Range 1–12)	7.45 ± 2.21 (3–12)	7.79 ± 2.01 (3–12)	7.65 ± 2.10 (3–12)
Reward scale (Range 1–28)	19.36 ± 3.63 (10–28)	19.82 ± 3.48 (7–28)	19.63 ± 3.55 (7–28)
ERI ratio	0.95 ± 0.38 (0.26–2.00)	0.95 ± 0.35 (0.28–3.67)	0.95 ± 0.36 (0.26–3.67)
> 1	76 (40.4)	92 (35.5)	168 (37.6)
≤ 1	112 (59.6)	167 (64.5)	279 (62.4)
**Resilience** (Range 0–40)	25.41 ± 5.73 (9–39)	27.19 ± 5.35 (8–39)	26.44 ± 5.58 (8–39)
**Depressive symptoms **(Range 0–30)	7.03 ± 4.74 (0–22)	4.88 ± 4.49 (0–20)	5.79 ± 4.71 (0–22)
no/ low symptoms (≤ 9)	137 (71.7)	221 (84.7)	358 (79.2)
minor depression (10–12)	30 (15.7)	19 (7.3)	49 (10.8)
major depression (≥ 13)	24 (12.6)	21 (8.0)	45 (10.0)

*Note*. *n* (%) or *M* ± *SD* (Range)

*hrs/w* hours per week, *LHD* lifetime history of depression, *WPC* work-privacy conflict, *ERI* effort-reward imbalance

^a^Multiple answers possible

^b^Subscale of the Copenhagen Psychosocial Questionnaire

Regarding the variables assessing psychosocial work stress, mean score was *M* = 38.15 (*SD* = 21.75) for WPC, *M* = 7.65 (*SD* = 2.10) for the ERI effort scale, and *M* = 19.63 (*SD* = 3.55) for the ERI reward scale. Mean ERI ratio was *M* = 0.95 (*SD* = 0.36), with 37.2% of all parents showing a ratio greater than 1, indicating that the perceived effort spent exceeded the reward. There were no significant gender differences regarding any of the indicators of psychosocial work stress. Mean resilience score was *M* = 26.44 (*SD* = 5.58), with mothers showing a lower score than fathers (*M*_mothers_ = 25.41, *M*_fathers_ = 27.19; *t*(426) = − 3.293, *p* = .001). The mean EPDS score was *M* = 5.79 (*SD* = 4.71) and 20.8% of participants had scores of at least 10, indicating at least a minor depression (Bergant et al., 1998; Cox et al., 1987). T-tests revealed that mothers had a significantly higher mean score than fathers (*M*_mothers_ = 7.03, *M*_fathers_ = 4.88; *t*(450) = 4.914, *p* < .001) and a higher percentage of scores indicating minor or major depression (*M*_mothers_ = 0.283, *M*_fathers_ = 0.153; *t*(450) = 3.385, *p* = .001). Of all parents, 12.6% reported a LHD.

### Correlational analysis

Results of the correlational analyses examining associations between confounders, predictors, moderator, and outcome can be found in Table 2. All indicators of psychosocial work stress showed significant associations with depressive symptoms (*p* < .001): Depressive symptoms were positively associated with the WPC score (*r* = .385) as well as with the effort scale of the ERI (*r* = .173) and the ERI ratio (*r* = .326). The ERI reward scale showed a negative association with depressive symptoms (*r* = −.338). WPC and the ERI ratio showed a moderate association (*r* = .458). Resilience showed significant negative associations with WPC, the ERI ratio, and depressive symptoms.

**Table 2:** Pearson correlation matrix including predictors, confounders, moderator, and outcome

Variable	1.	2.	3.	4.	5.	6.	7.	8.	9.
**1. Gender**	–								
**2. Working hrs/w**	.287^***^	–							
**3. LHD**	−.028	−.068	–						
**4. WPC**^a^	−.077	.029	.072	–					
**5. ERI effort**	.080	.193^***^	.050	.455^***^	–				
**6. ERI reward**	.065	.119^*^	−.105^*^	−.238^***^	−.165^***^	–			
**7. ERI ratio**	.012	.052	.088	.458^***^	.798^***^	−.650^***^	–		
**8. Resilience**	.158^**^	.075	−.219^***^	−.100^*^	−.098^*^	.225^***^	−.184^***^	–	
**9. Depressive symptoms**	−.226^***^	−.157^**^	.177^***^	.385^***^	.173^***^	−.338^***^	.326^***^	−.459^***^	–

*Note.* Sample size for analyses varied due to missing values of respective variables

*hrs/w* hours per week, *LHD* lifetime history of depression, *WPC* work-privacy conflict, *ERI* effort-reward imbalance

^a^Subscale of the Copenhagen Psychosocial Questionnaire

^*^p < .05. ^**^p < .01. ^***^p < .001

### Regression and moderation analysis

#### Work-privacy conflict

Detailed results of all models of the regression analyses including WPC are displayed in the supplement (see Supplementary Table 1, Additional file 2). Introducing only the confounders gender, working hours per week, and LHD in the first model explained 7.3% of the variance in depressive symptoms. Adding WPC as a predictor significantly improved *R*^*2*^ (Δ*R*^*2*^ = .132, *F*_1_, _417_ = 69.022, *p* < .001), as well as did adding resilience (Δ*R*^*2*^ = .142, *F*_1, 416_ = 91.540, *p* < .001) in the next step. In the last model including the interaction term, both WPC (β = 0.336, *p* < .001) and resilience (β = − 0.397, *p* < .001) remained significant predictors of depressive symptoms (Table 3), indicating that both a higher WPC and lower resilience are associated with stronger depressive symptoms. However, this model did not show any additional explanatory value (Δ*R*^*2*^ = .003, *F*_1, 415_ = 1.137, *p* = .167) and the interaction term did not become significant (β = 0.055, *p* = .167). Only gender was a significant confounder in the last model (β = − 0.089, *p* = .034). The final model accounted for 35.1% of variance in depressive symptoms.

**Table 3:** Multiple linear regression of depressive symptoms on indicators of psychosocial work stress (WPC, ERI), resilience, and their interaction, controlled for potential confounders

	***B***	***SE B***	β	95% CI		***p***	***R***^***2***^
				upper	lower		
**Model including WPC**							.351
Constant	7.183	0.610		6.019	8.495	.000	
Gender	−0.841	0.394	**−0.089**	−1.551	−0.180	.034	
Working hrs/ week	−0.030	0.016	−0.079	−0.062	0.003	.056	
LHD	0.804	0.684	0.058	−0.506	2.156	.155	
WPC^a,b^	0.073	0.010	** 0.336**	0.053	0.092	.000	
Resilience^b^	−0.334	0.042	**−0.397**	−0.412	−0.246	.000	
WPC^a,b^ x resilience^b^	0.002	0.002	0.055	−0.001	0.006	.167	
**Model including ERI ratio**							.308
Constant	7.373	0.618		6.083	8.673	.000	
Gender	−1.118	0.400	**−0.117**	−1.961	−0.319	.007	
Working hrs/ week	−0.036	0.017	**−0.092**	−0.068	−0.004	.031	
LHD	0.872	0.683	0.063	−0.518	2.218	.139	
ERI ratio^b^	3.041	0.552	**0.254**	1.918	4.104	.000	
Resilience^b^	−0.315	0.039	**−0.371**	−0.391	−0.230	.000	
ERI ratio^b^ x resilience^b^	−0.203	0.102	**−0.101**	−0.418	0.005	.018	

*Note. *Standard errors of *B* and 95% bias corrected and accelerated confidence intervals are based on 1000 bootstrap samples.

*WPC* work-privacy conflict, *LHD* lifetime history of depression, *ERI* effort-reward imbalance.

^a^Subscale of the Copenhagen Psychosocial Questionnaire

^b^Mean-centered

#### Effort-reward imbalance

Detailed results of all models of the regression analysis including the ERI ratio are displayed in the supplement (see Supplementary Table 2, Additional file 2). The confounders entered in the first model explained 7.6% of the variance in depressive symptoms. Adding the log-transformed ERI ratio as a predictor significantly improved *R*^*2*^ (Δ*R*^*2*^ = 0.097, *F*_1, 414_ = 48.644, *p* < .001), as well as did adding resilience in the next step (Δ*R*^*2*^ = 0.125, *F*_1, 413_ = 73.713, *p* < .001). In the last model including the interaction term (Table 3), both the ERI ratio (β = 0.254, *p* < .001) and resilience (β = − 0.371, *p* < .001) remained significant predictors of depressive symptoms. The interaction term became significant as well (β = − 0.101, *p* = .018), however, the bias corrected and accelerated 95% CI for *B* was [− 0.418, 0.005] including zero. The final model explained 30.8% of variance in depressive symptoms (Δ*R*^*2*^ = 0.010, *F*_1, 414_ = 5.668, *p* = .018), with gender (β = − 0.117, *p* = .007) as well as working hours per week (β = − 0.092, *p* = .031) both being significant confounders.

### Post-hoc analysis

**Fig. 2:**
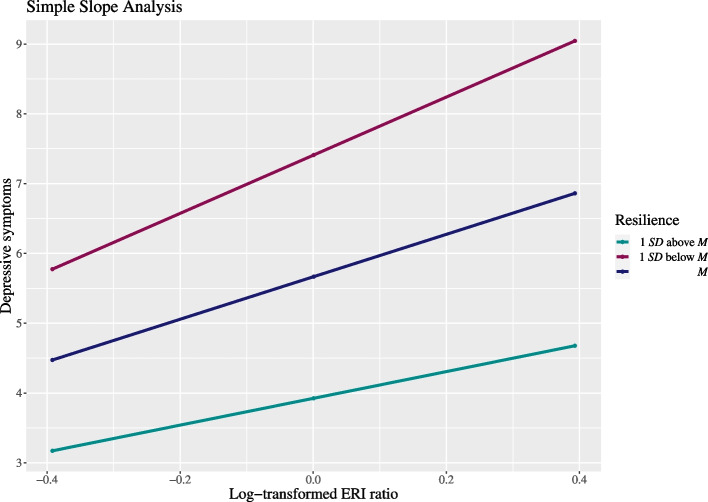
Simple slopes of the log-transformed ERI ratio predicting depressive symptoms*. Note.* EPDS = Edinburgh Postnatal Depression Scale. ERI = effort-reward imbalance. CD-RISC = Connor-Davidson Resilience Scale. *SD* = standard deviation. *M* = mean

To further investigate the moderating role of resilience on the relationship between the ERI ratio and depressive symptoms, a simple slope analysis was conducted using the SPSS tool PROCESS [67] (Fig. 2). The unstandardized simple slope for a mean resilience score was *B* = 3.041 (95% CI [2.627, 5.703], *t* = 5.328, *p* < .001). If the resilience score was 1 *SD* below and 1 *SD* above the mean of resilience, the unstandardized slope was *B* = 4.165 (95% CI [2.016, 4.067], *t* = 5.830, *p* < .001) and *B* = 1.917 (95% CI [0.7073, 3.1272], *t* = 3.115, *p* = .002), respectively.

## Discussion

The aim of this study was to examine the association between psychosocial work stress and depressive symptoms in working parents during the COVID-19 pandemic on the one hand and to investigate whether this relationship was moderated by resilience on the other hand. Previous studies have found that work-related factors can be risk factors for parents’ mental health in general [14, 71]. The current study provides a more differentiated insight into the nature of possible work-related risk factors as specifically WPC and ERI were investigated as indicators of psychosocial work stress. Beyond that, the potential protective role of individual resilience was examined.

Analyses revealed that higher WPC may be associated with more depressive symptoms in working parents during the COVID-19 pandemic. The potential of WPC as a risk factor is in line with previous findings from pre-pandemic studies [34, 36, 72]. To our knowledge, no other study has investigated this association in a sample of working parents during the pandemic so far, but a few studies found results supporting the relevance of WPC regarding mental health: López and colleagues [73] found WPC to be a significant predictor of depression in the general population while Meyer and colleagues [18] identified WPC to be associated with exhaustion in a sample of employees. These findings support the assumption that especially during the pandemic, interference of work with private life needs to be considered as a risk factor for mental health. With the majority of parents in our study being required to work from home as a consequence of the pandemic, integrating work at home is likely to cause blurry boundaries between family and work context, requiring several role transitions within one day and increasing the risk for experiencing a conflict between the two domains during the pandemic [74].

Regarding the buffering role of resilience on the association between WPC and depressive symptoms, our hypothesis could not be confirmed. High resilience did not weaken the association between WPC and depressive symptoms. This could be due to different reasons. First, resilience can be seen as a process of adaptation [75, 76], therefore requiring time. Hence, our results might not have revealed a buffering effect of resilience on the association between WPC and depressive symptoms because the process of adaptation had still been ongoing. The COVID-19 pandemic represents a novel situation in which aspects such as the time it takes to adapt to the drastic changes working parents were confronted with, still need to be explored. Therefore, longitudinal studies with assessments at a later stage should further investigate the moderating role of resilience, which had not yet unfolded at the time of the first DREAM_CORONA_ assessment. Second, this study only investigated resilience at an individual level. Resilience however can be context-dependent [77], and WPC clearly involves the context of one’s family and workplace. Therefore, other protective resilience factors like family resilience [78] or social support provided by supervisors, colleagues, spouses, or family [79, 80] might be more crucial in this context than individual resilience. Third, there are different conceptual models of resilience, elaborating the way resilience might affect mental health and well-being [81]. In the compensatory model, resilience can exert its influence as a promoting factor, counteracting the exposure to risk by directly affecting the outcome [77, 81, 82]. In the protective factor model, resilience is assumed to moderate the effect of a present risk on the outcome [77, 81, 82]. The latter mechanism was investigated in the present study. However, as resilience can be context-dependent [77], WPC might represent a situation in which resilience rather relates to mental health as a promoting factor as suggested in the compensatory model, thereby counteracting the negative association between WPC and mental health. This would be in line with our findings as higher individual resilience was strongly associated with lower depressive symptoms when WPC was included in the regression model as well.

As hypothesized, ERI also showed a significant association with depressive symptoms in working parents during the pandemic, which is in line with results of pre-pandemic studies [39, 83, 84]. Findings with reference to the pandemic are limited. However, Magnavita and colleagues [85] reported a similar association of the ERI ratio with depressive symptoms in a sample of employees. As elaborated above, according to the ERI model [38], effort refers to meeting obligations and demands while rewards can either be received as financial rewards (e.g., salary), status-related rewards (e.g., career promotion or job security), or social-emotional reward (e.g., esteem or recognition). In the context of the pandemic, adaptation to changes in demands and obligations at work due to political restrictions might be perceived as high effort spent on work (e.g., demands regarding sudden adjustments to working from home if no adequate infrastructure was in place yet). Employees were required to adapt to new technologies and ways to collaborate and were forced to limit personal contact with co-workers. At the same time receiving a reward might have been scarce: The economic crisis might threaten job security or promotion while limited personal contact and face-to-face interaction might diminish possibilities to receive acknowledgment of one’s work by co-workers or supervisors [2, 4, 86].

Regarding the buffering effect of individual resilience on the association between ERI and depressive symptoms, a significant moderation was found. The simple slope analysis illustrates the effect (Fig. 2), suggesting that in working mothers and fathers with greater individual resilience the negative association between ERI and depressive symptoms might be weaker than in those parents with lower individual resilience. However, the effect was only marginally significant, and the bias corrected and accelerated bootstrap confidence interval included zero, suggesting non-significance. Therefore, this result needs to be interpreted with caution. So far, no study has investigated the buffering effect of resilience on the relationship of ERI and depressive symptoms, neither before nor after the outbreak of the pandemic. However, Havnen and colleagues [50] reported that resilience moderated the effect of exposure to perceived stress on depressive symptoms during the COVID-19 pandemic, which supports the tendency of our findings.

Apart from the presence of psychosocial work stress in parents during the pandemic, the observed prevalence of depressive symptoms in our sample raises concerns. Overall, the sample showed a high prevalence of EPDS scores indicating minor or major depression (20.8%). This is in line with other studies conducted during the pandemic, which indicated both an elevation in maternal depression [15] as well as an elevation in self-reported depressive symptoms in a sample of the general population [87]. Moreover, increased clinically significant levels of mental distress [17] and a lower well-being of parents, especially of parents living with younger children and of women were reported [88]. The latter finding supports the observed gender differences regarding depressive symptoms in our study as mothers had a higher mean of depressive symptoms compared to fathers. This difference is common in data independent of the pandemic situation, as women have a higher risk of suffering from depression in their lifetime [89]. Hence, it is not surprising that gender was a significant confounder in all regression analyses. Along with gender differences regarding depressive symptoms, we also found significant gender differences regarding resilience, with fathers showing greater resilience compared to mothers. This is in line with the finding that greater resilience showed a strong negative association with depressive symptoms. No gender differences were found for indicators of psychosocial work stress.

Regarding the other control variables LHD and working hours per week, only the latter was a significant confounder in the final model investigating the association between ERI and depressive symptoms. This result indicates that working more hours was related to less depressive symptoms which is in line with findings of Witteveen and Velthorst [90]. They reported that a sudden decreased workload during the pandemic was associated with greater feelings of depression as compared to workers whose workload remained stable [90]. This effect could be explained by a decrease of workload being accompanied by financial strains or short-time work, thereby eliciting stress, which in turn might affect feelings of depression. Furthermore, being forced to work less hours might threaten one’s daily structure or routine, which could normally benefit mental health [91]. However, as our cross-sectional design does not allow conclusions regarding causality, the observed association might as well suggest that better mental health enables employees to work longer hours.

### Strengths and limitations

To the best of our knowledge, this study was the first to examine the association between specific psychosocial work factors and depressive symptoms in working parents during the COVID-19 pandemic and to investigate the buffering effect of individual resilience on this association. This was possible both for mothers and fathers. In addition, participants had at least one young child aged 0 to 34 months offering valuable insights into the mental health of parents of young children. The DREAM_CORONA_ study is part of a prospective longitudinal cohort study [53] and therefore allows to build on the present findings in future assessments. As the dynamic of the pandemic required a quick response to grasp the ongoing processes during the pandemic, cross-sectional data of the first assessment were analysed as a start in order to gain a first understanding. As another major strength, our study contributes to a more comprehensive picture of potential factors promoting mental health. This is particularly valuable considering the concept of positive psychology and the importance of strengthening psychological resources in order to prevent mental disorders in the first place [92, 93].

At the same time, there are some limitations to be considered when interpreting the present results. First, the characteristics of the study sample prevent us from transferring the results to working parents in general. Our sample consisted of highly educated mothers and fathers, which is not representative for the general population. However, this is not unusual for epidemiological studies [94] and there was no significant association of parental education with the outcome, i.e., depressive symptoms, in our sample. Moreover, almost all participants were in a permanent relationship, and most of them had only one child. Therefore, psychosocial work stress, resilience, and depressive symptoms might be different in single parents or those with more children. Second, the data of the present sample of the DREAM_CORONA_ study were derived from a cross-sectional assessment and did not include a pre-pandemic baseline. Therefore, no causal interpretations can be made whether any values of our research variables have increased or decreased compared to the time prior to the outbreak. However, pre-pandemic depression prevalences in different subsamples from the general DREAM study give reason to assume that depressive symptoms are elevated during the pandemic [95–97]. Third, as the pandemic is a highly dynamic process with permanent changes regarding political restrictions and work, social, and family life, the findings of the present study explicitly refer to early stages of the pandemic, assessed from May to June 2020. This implicates that a comparison with studies conducted under non-pandemic circumstances is limited. Fourth, by calculating a ratio of effort and reward, a linear association between effort and reward is assumed thereby reducing complexity. However, this might not reflect the whole nature of this association which might only be partially linear as a comparatively higher amount of perceived effort may be required for an increase of the perceived reward or vice versa at a certain point. Fifth, data were based on self-report only and therefore might be susceptible to biases such as social desirability. However, we only used validated instruments, which are widely used in research.

### Implications and future research

Our findings have two major implications. First, as both ERI and WPC were associated with poorer mental health, measures are needed to decrease psychosocial work stress. Second, given the potential of individual resilience to buffer the association between psychosocial work stress and depressive symptoms on the one hand and to directly benefit mental health on the other hand, resilience needs to be fostered. Decreasing psychosocial work stress could be achieved by fostering family-friendly organizational conditions (e.g., supportive organizational climate, flexitime, or other arrangements to autonomously manage work demands), which have been shown to diminish WPC [98]. Apart from structural measures, individuals themselves should be supported to cope with WPC, e.g., through enhancing to manage boundaries between work and family as low boundary management has been shown to be associated with higher WPC [99]. Identifying ways to increase rewards at work during the pandemic could reduce the imbalance of effort and reward. A possible measure which might enhance rewards like job security or recognition could be establishing an adjusted organizational communication, which transmits transparency about the pandemic dynamic, actively involves the employees, and strengthens a feeling of belonging [100]. Moreover, receiving higher rewards might be a protective factor itself, as higher compared to lower perceived reward is associated with a lower risk for depression [84]. In order to foster resilience, health insurances as well as employers should provide targeted resilience trainings as interventions have been proven to effectively increase resilience both in general and in contexts of occupational stress [101–103]. Recalling the role of parents’ mental health in family stress models, reducing psychosocial work stress and strengthening resilience appear even more important in order to prevent the negative effects of poor mental health of parents on both parenting and children’s well-being.

Regarding future research, studies should investigate the potential protective role of resilience at a later stage of the pandemic to examine whether its buffering effect unfolds more strikingly after a longer period of adjustment. In addition, longitudinal studies are needed to draw conclusions regarding the predictive value of WPC, ERI, and resilience on depressive symptoms in working parents. Beyond that, the investigation of potential protective factors buffering the effect of psychosocial work stress should be broadened, particularly with regard to other potential resilience factors, which might be associated with WPC (e.g., family resilience, social support at home or at work). In addition, positive effects of restrictions due to the pandemic (e.g., no commuting to work, spouses being able to mutually support each other at home) should be investigated.

## Conclusions

The findings of the present study advance the current knowledge about the mental health status of working parents during the COVID-19 pandemic and potential risk and protective factors. Higher psychosocial work stress was significantly associated with more depressive symptoms, while greater resilience was associated with less depressive symptoms. Beyond that, the findings of the present study partially support the protective role of resilience buffering the association between psychosocial stress and mental health. Our findings highlight the importance to reduce psychosocial work stress and to foster resilience in order to promote mental health in working parents during the pandemic and thereby ultimately benefiting the mental health of the entire family.

## Supplementary Information

**Additional file 1.** Flowchart of participation rate and exclusion criteria resulting in final sample. The flowchart illustrates the stepwise applied exclusion criteria (no consent, respondance outside the given time frame, not having children yet, and no current employment, incomplete data).

**Additional file 2.** Full results of multiple regression analysis. The included tables (Supplementary Table 1 and 2) show the full results of the multiple regression analysis of depressive symptoms on psychosocial work stress, resilience, and their interaction, while controlling for potential confounders.

## Data Availability

The dataset analysed during the current study is not publicly available due to legal and ethical constraints. Public sharing of participant data was not included in the informed consent of the study. All enquiries about access to data should be sent to the corresponding author. All requests to access data will be handled in accordance with the Ethics Committee of the Faculty of Medicine of the Technische Universität Dresden.

## Abbreviations

CI: confidence interval
CD-RISC: Connor-Davidson Resilience Scale
DREAM: Dresden Study on Parenting, Work, and Mental Health (“DResdner Studie zu Elternschaft, Arbeit und Mentaler Gesundheit”)
EPDS: Edinburgh Postnatal Depression Scale
ERI: effort-reward imbalance
LHD: lifetime history of depression
WHO: World Health Organization
WPC: work-privacy conflict

## References

[1] World Health Organization. Coronavirus disease 2019 (COVID-19) situation report – 51. Geneva; 2020 [cited 2020 Nov 20]. Available from: https://www.who.int/docs/default-source/coronaviruse/situation-reports/20200311-sitrep-51-covid-19.pdf?sfvrsn=1ba62e57_10.

[2] Frodermann C, Grunau P, Haepp T, Mackeben J, Ruf K, Steffes S (2020). Online-Befragung von Beschäftigten: Wie Corona den Arbeitsalltag verändert hat. IAB-Kurzbericht 13.

[3] Möhring K, Naumann E, Reifenscheid M, Wenz A, Rettig T, Krieger U (2021). The COVID-19 pandemic and subjective well-being: longitudinal evidence on satisfaction with work and family. Eur Soc.

[4] Rigotti T, De Cuyper N, Sekiguchi T (2020). The Corona crisis: what can we learn from earlier studies in applied psychology?. Appl Psychol.

[5] Müller K-U, Samtleben C, Schmieder J, Wrohlich K (2020). Corona-Krise erschwert Vereinbarkeit von Beruf und Familie vor allem für Mütter - Erwerbstätige Eltern sollten entlastet werden. DIW Wochenbericht.

[6] Deutsches Jugendinstitut, Robert-Koch-Institut. Monatsbericht der Corona-KiTa-Studie. Monatsbericht der Corona-KiTa-Studie - Mai 2020. 2020 [cited 2020 Nov 21]. Available from: https://www.rki.de/DE/Content/InfAZ/N/Neuartiges_Coronavirus/Projekte_RKI/KiTa-Studie-Berichte/KiTAStudie_05_2020.pdf?__blob=publicationFile

[7] Fisher J, Languilaire JC, Lawthom R, Nieuwenhuis R, Petts RJ, Runswick-Cole K (2020). Community, work, and family in times of COVID-19. Community Work Fam.

[8] Ammar A, Mueller P, Trabelsi K, Chtourou H, Boukhris O, Masmoudi L (2020). Psychological consequences of COVID-19 home confinement: the ECLB-COVID19 multicenter study. PLoS One.

[9] Petzold MB, Bendau A, Plag J, Pyrkosch L, Mascarell Maricic L, Betzler F (2020). Risk, resilience, psychological distress, and anxiety at the beginning of the COVID-19 pandemic in Germany. Brain Behav.

[10] Xiong J, Lipsitz O, Nasri F, Lui LMW, Gill H, Phan L (2020). Impact of COVID-19 pandemic on mental health in the general population: a systematic review. J Affect Disord.

[11] Horiuchi S, Shinohara R, Otawa S, Akiyama Y, Ooka T, Kojima R (2020). Caregivers’ mental distress and child health during the COVID-19 outbreak in Japan. PLoS One.

[12] Marchetti D, Fontanesi L, Mazza C, Di Giandomenico S, Roma P, Verrocchio MC (2020). Parenting-related exhaustion during the italian COVID-19 lockdown. J Pediatr Psychol.

[13] Patrick SW, Henkhaus LE, Zickafoose JS, Lovell K, Halvorson A, Loch S (2020). Well-being of parents and children during the COVID-19 pandemic: a national survey. Pediatrics..

[14] Cusinato M, Iannattone S, Spoto A, Poli M, Moretti C, Gatta M (2020). Stress, resilience, and well-being in Italian children and their parents during the COVID-19 pandemic. Int J Environ Res Public Health.

[15] Cameron EE, Joyce KM, Delaquis CP, Reynolds K, Protudjer JLP, Roos LE (2020). Maternal psychological distress & mental health service use during the COVID-19 pandemic. J Affect Disord.

[16] Huebener M, Spieß CK, Siegel N, Wagner G (2020). Wohlbefinden von Familien in Zeiten von Corona: Eltern mit jungen Kindern am stärksten beeinträchtigt. DIW Wochenbericht.

[17] Pierce M, Hope H, Ford T, Hatch S, Hotopf M, John A (2020). Mental health before and during the COVID-19 pandemic: a longitudinal probability sample survey of the UK population. Lancet Psychiatry.

[18] Meyer B, Zill A, Dilba D, Gerlach R, Schumann S (2021). Employee psychological well-being during the COVID-19 pandemic in Germany: a longitudinal study of demands, resources, and exhaustion. Int J Psychol.

[19] Romero E, López-Romero L, Domínguez-Álvarez B, Villar P, Gómez-Fraguela JA (2020). Testing the effects of covid-19 confinement in spanish children: the role of parents’ distress, emotional problems and specific parenting. Int J Environ Res Public Health.

[20] Goodman SH, Rouse MH, Connell AM, Broth MR, Hall CM, Heyward D (2011). Maternal depression and child psychopathology: a meta-analytic review. Clin Child Fam Psychol Rev.

[21] Vostanis P, Graves A, Meltzer H, Goodman R, Jenkins R, Brugha T (2006). Relationship between parental psychopathology, parenting strategies and child mental health: findings from the GB national study. Soc Psychiatry Psychiatr Epidemiol.

[22] Morelli M, Cattelino E, Baiocco R, Trumello C, Babore A, Candelori C (2020). Parents and children during the COVID-19 lockdown: the influence of parenting distress and parenting self-efficacy on children’s emotional well-being. Front Psychol.

[23] Conger RD, Donnellan MB (2007). An interactionist perspective on the socioeconomic context of human development. Annu Rev Psychol.

[24] Masarik AS, Conger RD (2017). Stress and child development: a review of the family stress model. Curr Opin Psychol.

[25] Belsky J (1984). The determinants of parenting: a process model. Child Dev.

[26] Neppl TK, Senia JM, Donnellan MB (2016). The effects of economic hardship: testing the family stress model over time. J Fam Psychol.

[27] Lieb R, Isensee B, Höfler M, Pfister H, Wittchen HU (2002). Parental major depression and the risk of depression and other mental disorders in offspring: a prospective-longitudinal community study. Arch Gen Psychiatry.

[28] Psychogiou L, Moberly NJ, Parry E, Nath S, Kallitsoglou A, Russell G (2017). Parental depressive symptoms, children’s emotional and behavioural problems, and parents’ expressed emotion - critical and positive comments. PLoS One.

[29] Conger RD, Conger KJ (2002). Resilience in midwestern families: selected findings from the first decade of a prospective, longitudinal study. J Marriage Fam.

[30] Eurofound, European Agency for Safety and Health at work (2014). Psychosocial risks in Europe - prevalence and strategies for prevention: a joint report from the European Foundation for the Improvement of living and working conditions and the European Agency for Safety and Health at work.

[31] Staines GL (1980). Spillover versus compensation: a review of the literature on the relationship between work and nonwork. Hum Relat.

[32] Eby LT, Maher CP, Butts MM (2010). The intersection of work and family life: the role of affect. Annu Rev Psychol.

[33] Amstad FT, Meier LL, Fasel U, Elfering A, Semmer NK (2011). A meta-analysis of work-family conflict and various outcomes with a special emphasis on cross-domain versus matching-domain relations. J Occup Health Psychol.

[34] Frone MR, Russell M, Cooper ML (1997). Relation of work-family conflict to health outcomes: a four-year longitudinal study of employed parents. J Occup Organ Psychol.

[35] Garthus-Niegel S, Hegewald J, Seidler A, Nübling M, Espinola-Klein C, Liebers F (2016). The Gutenberg health study : associations between occupational and private stress factors and work-privacy conflict. BMC Public Health.

[36] Hämmig O, Gutzwiller F, Bauer G (2009). Work-life conflict and associations with work- and nonwork-related factors and with physical and mental health outcomes: a nationally representative cross-sectional study in Switzerland. BMC Public Health.

[37] Siegrist J (1996). Adverse health effects of high-effort/ low-reward conditions. J Occup Health Psychol.

[38] Siegrist J, Cooper CL, Campbell Quick J (2017). The effort-reward imbalance model. The handbook of stress and health - a guide to research and practice.

[39] Rugulies R, Aust B, Madsen IEH (2017). Effort–reward imbalance at work and risk of depressive disorders. A systematic review and meta-analysis of prospective cohort studies. Scand J Work Environ Health.

[40] van der Molen HF, Nieuwenhuijsen K, Frings-Dresen MHW, de Groene G (2020). Work-related psychosocial risk factors for stress-related mental disorders: an updated systematic review and meta-analysis. BMJ Open.

[41] Magnavita N, Maurizio Soave P, Ricciardi W, Antonelli M (2020). Occupational stress and mental health among anesthetists during the COVID-19 pandemic. Int J Environ Res Public Health.

[42] Zhang J, Wang Y, Xu J, You H, Li Y, Liang Y (2021). Prevalence of mental health problems and associated factors among front-line public health workers during the COVID-19 pandemic in China: an effort–reward imbalance model-informed study. BMC Psychol.

[43] Bengel J, Lyssenko L (2012). Resilienz und psychologische Schutzfaktoren im Erwachsenenalter - Stand der Forschung zu psychologischen Schutzfaktoren von Gesundheit im Erwachsenenalter. Forschung und Praxis der Gesundheitsförderung Köln: Bundeszentrale für gesundheitliche Aufklärung..

[44] Davydov DM, Stewart R, Ritchie K, Chaudieu I (2010). Resilience and mental health. Clin Psychol Rev.

[45] Rutten BPF, Hammels C, Geschwind N, Menne-Lothmann C, Pishva E, Schruers K (2013). Resilience in mental health: linking psychological and neurobiological perspectives. Acta Psychiatr Scand.

[46] Noeker M, Petermann F (2008). Resilienz: Funktionale Adaptation an widrige Umgebungsbedingungen. Z Psychiatr Psychol Psychother.

[47] Hu T, Zhang D, Wang J (2015). A meta-analysis of the trait resilience and mental health. Personal Individ Differ.

[48] Gloria CT, Steinhardt MA (2016). Relationships among positive emotions, coping, resilience and mental health. Stress Health.

[49] Sheerin CM, Lind MJ, Brown EA, Gardner CO, Kendler KS, Amstadter AB (2018). The impact of resilience and subsequent stressful life events on MDD and GAD. Depress Anxiety.

[50] Havnen A, Anyan F, Hjemdal O, Solem S, Riksfjord Gurigard M, Hagen K (2020). Resilience moderates negative outcome from stress during the COVID-19 pandemic: a moderated-mediation approach. Int J Environ Res Public Health.

[51] Kinser PA, Jallo N, Amstadter AB, Thacker LR, Jones E, Moyer S (2021). Depression, anxiety, resilience, and coping: the experience of pregnant and new mothers during the first few months of the COVID-19 pandemic. J Women's Health.

[52] Lenzo V, Quattropani MC, Musetti A, Zenesini C, Freda MF, Lemmo D, et al. Resilience contributes to low emotional impact of the COVID-19 outbreak among the general population in Italy. Front Psychol. 2020:11. 10.3389/fpsyg.2020.576485. 10.3389/fpsyg.2020.576485 PMC7672208 33250818

[53] Kress V, Steudte-Schmiedgen S, Kopp M, Förster A, Altus C, Schier C (2019). The impact of parental role distributions, work participation, and stress factors on family health-related outcomes: study protocol of the prospective multi-method cohort “Dresden study on parenting, work, and mental health” (DREAM). Front Psychol.

[54] Kristensen TS, Hannerz H, Høgh A, Borg V (2005). The Copenhagen psychosocial questionnaire - a tool for the assessment and improvement of the psychosocial work environment. Scand J Work Environ Health.

[55] Nübling M, Stößel U, Hasselhorn H, Michaelis M, Hofmann F (2005). Methoden zur Erfassung psychischer Belastungen - Erprobung eines Messinstrumentes (COPSOQ).

[56] Rödel A, Siegrist J, Hessel A, Brähler E (2004). Fragebogen zur Messung beruflicher Gratifikationskrisen. Zeitschrift für Differ und Diagnostische Psychol.

[57] Siegrist J, Wege N, Pühlhofer F, Wahrendorf M (2009). A short generic measure of work stress in the era of globalization: effort-reward imbalance. Int Arch Occup Environ Health.

[58] Siegrist J, Starke D, Chandola T, Godin I, Marmot M, Niedhammer I (2004). The measurement of effort-reward imbalance at work: European comparisons. Soc Sci Med.

[59] Niedhammer I, Tek ML, Starke D, Siegrist J (2004). Effort-reward imbalance model and self-reported health: cross-sectional and prospective findings from the GAZEL cohort. Soc Sci Med.

[60] Pikhart H, Bobak M, Siegrist J, Pajak A, Rywik S, Kyshegyi J (2001). Psychosocial work characteristics and self rated health in four post-communist countries. J Epidemiol Community Health.

[61] Connor KM, Davidson JRT (2003). Development of a new resilience scale: the Connor-Davidson resilience scale (CD-RISC). Depress Anxiety.

[62] Bergant AM, Nguyen T, Heim K, Ulmer H, Dapunt O (1998). Deutschsprachige Fassung und Validierung der “Edinburgh Postnatal Depression Scale.”. Dtsch Med Wochenschr.

[63] Cox JL, Holden JM, Sagovsky R (1987). Detection of postnatal depression: development of the 10-item Edinburgh postnatal depression scale. Br J Psychiatry.

[64] Cox JL, Chapman G, Murray D, Jones P (1996). Validation of the Edinburgh postnatal depression scale (EPDS) in non-postnatal women. J Affect Disord.

[65] Massoudi P, Hwang CP, Wickberg B (2013). How well does the Edinburgh postnatal depression scale identify depression and anxiety in fathers? A validation study in a population based Swedish sample. J Affect Disord.

[66] Weigl T, Garthus-Niegel S (2021). Questionnaires for the assessment of Peripartum depression, anxiety and stress (part 1 of a series on psychological assessment during the peripartum period). Z Geburtshilfe Neonatol.

[67] Hayes AF (2017). Introduction to mediation, moderation, and conditional process analysis: A regressionbased approach.

[68] Field A (2018). Discovering statistics using SPSS.

[69] Statistisches Landesamt Sachsen. Mikrozensusergebnisse: Bevölkerung nach Schulabschluss und Berufsabschluss. 2020 [cited 2020 Nov 20]. Available from: https://www.dresden.de/de/leben/stadtportrait/statistik/bevoelkerung-gebiet/mikrozensus.php.

[70] Keller M, Kahle I. Realisierte Erwerbstätigkeit von Müttern und Vätern zur Vereinbarkeit von Familie und Beruf. WISTA. 2018;3 [cited 2020 Nov 21]. Available from: http://www.destatis.de/DE/Methoden/WISTA-Wirtschaft-und-Statistik/2018/03/realisierte-erwerbstaetigkeit-032018.pdf?__blob=publicationFile.

[71] Broadway B, Mendez S, Moschion J. Behind closed doors: The surge in mental distress of parents. 2020. [cited 2020 Nov 20]. Available from: https://findanexpert.unimelb.edu.au/scholarlywork/1486519-behind-closed-doors%2D%2Dthe-surge-in-mental-distress-of-parents.

[72] Wang J, Smailes E, Sareen J, Schmitz N, Fick G, Patten S (2012). Three job-related stress models and depression: a population-based study. Soc Psychiatry Psychiatr Epidemiol.

[73] López-Núñez MI, Díaz-Morales JF, Aparicio-García ME (2021). Individual differences, personality, social, family and work variables on mental health during COVID-19 outbreak in Spain. Personal Individ Differ.

[74] Ashforth BE, Kreiner GE, Fugate M (2000). All in a day’s work: boundaries and micro role transitions. Acad Mangement Rev.

[75] Fletcher D, Sarkar M (2013). Psychological resilience: a review and critique of definitions, concepts, and theory. Eur Psychol.

[76] Ungar M, Theron L (2019). Resilience and mental health: how multisystemic processes contribute to positive outcomes. Lancet Psychiatry.

[77] Fergus S, Zimmerman MA (2005). Adolescent resilience: a framework for understanding healthy development in the face of risk. Annu Rev Public Health.

[78] Grzywacz JG, Bass BL (2003). Work, family, and mental health: testing different models of work-family fit. J Marriage Fam.

[79] Etzion D (1984). Moderating effect of social support on the stress-burnout relationship. J Appl Psychol.

[80] Kossek EE, Pichler S, Bodner T, Hammer LB (2011). Workplace social support and work-family conflict: a meta-analysis clarifying the influence of general and work-family-specific supervisor and organizational support. Pers Psychol.

[81] Zimmerman MA, Stoddard SA, Eisman AB, Caldwell CH, Aiyer SM, Miller A (2013). Adolescent resilience: Promotive factors that inform prevention. Child Dev Perspect.

[82] Rutter M (1985). Resilience in the face of adversity: protective factors and resistance to psychiatric disorder. Br J Psychiatry.

[83] Nigatu YT, Wang J (2018). The combined effects of job demand and control, effort-reward imbalance and work-family conflicts on the risk of major depressive episode: a 4-year longitudinal study. Occup Environ Med.

[84] Wege N, Li J, Siegrist J (2018). Are there gender differences in associations of effort-reward imbalance at work with self-reported doctor-diagnosed depression? Prospective evidence from the German socio-economic panel. Int Arch Occup Environ Health.

[85] Magnavita N, Tripepi G, Chiorri C (2021). Telecommuting, off-time work, and intrusive leadership in workers’ well-being. Int J Environ Res Public Health.

[86] Lippens L, Moens E, Sterkens P, Weytjens J, Baert S (2021). How do employees think the COVID-19 crisis will affect their careers?. PLoS One.

[87] Bäuerle A, Steinbach J, Schweda A, Beckord J, Hetkamp M, Weismüller B (2020). Mental health burden of the COVID-19 outbreak in Germany: predictors of mental health impairment. J Prim Care Community Health.

[88] Huebener M, Waights S, Spiess CK, Siegel NA, Wagner GG (2021). Parental well-being in times of Covid-19 in Germany. Rev Econ Househ.

[89] Kuehner C (2016). Why is depression more common among women than among men?. Lancet Psychiatry.

[90] Witteveen D, Velthorst E (2020). Economic hardship and mental health complaints during COVID-19. Proc Natl Acad Sci.

[91] Baumann A, Muijen M, Gaebel W. Mental health and well-being at the workplace – protection and inclusion in challenging times. World health. Organization. 2010; [cited 2022 Jan 9]. Available from: https://www.euro.who.int/en/publications/abstracts/mental-health-and-well-being-at-the-workplace-protection-and-inclusion-in-challenging-times-2010.

[92] Yates TM, Tyrell F, Masten AS, Joseph S (2014). Resilience theory and the practice of positive psychology from individuals to societies. Positive psychology in practice. Second.

[93] Kobau R, Seligman MEP, Peterson C, Diener E, Zack MM, Chapman D (2011). Mental health promotion in public health: perspectives and strategies from positive psychology. Am J Public Health.

[94] Søgaard AJ, Selmer R, Bjertness E, Thelle D. The Oslo health study: the impact of self-selection in a large, population-based survey. Int J Equity Health. 2004;3(3). 10.1186/1475-9276-3-3. 10.1186/1475-9276-3-3 PMC428581 15128460

[95] Garthus-Niegel S, Staudt A, Kinser P, Haga SM, Drozd F, Baumann S (2020). Predictors and changes in paternal perinatal depression profiles — insights from the DREAM study. Front Psychiatry.

[96] Schaber R, Karl M, Kopp M, Kress V, Weidner K, Martini J (2020). My job, my child, my house: the predictive value of job- and housework-related factors on depressive symptoms during the postpartum period. J Affect Disord.

[97] Karl M, Schaber R, Kress V, Kopp M, Martini J, Weidner K (2020). Precarious working conditions and psychosocial work stress act as a risk factor for symptoms of postpartum depression during maternity leave: results from a longitudinal cohort study. BMC Public Health.

[98] Selvarajan TT, Cloninger PA, Singh B (2013). Social support and work- family conflict: a test of an indirect effects model. J Vocat Behav.

[99] Kossek E, Ruderman MN, Braddy PW, Hannum KM (2012). Work-nonwork boundary management profiles: a person-centered approach. J Vocat Behav.

[100] Zito M, Ingusci E, Cortese CG, Giancaspro ML, Manuti A, Molino M, et al. Does the end justify the means? The role of organizational communication among work-from-home employees during the COVID-19 pandemic. Int J Environ Res Publich Heal. 2021;18(3933). 10.3390/ijerph18083933. 10.3390/ijerph18083933 PMC8069567 33918095

[101] Babanataj R, Mazdarani S, Hesamzadeh A, Gorji MH, Cherati JY (2019). Resilience training: effects on occupational stress and resilience of critical care nurses. Int J Nurs Pract.

[102] Steinhardt M, Dolbier C (2008). Evaluation of a resilience intervention to enhance coping strategies and protective factors and decrease symptomatology. J Am Coll Heal.

[103] Chitra T, Karunanidhi S (2021). The impact of resilience training on cccupational stress, resilience, job satisfaction, and psychological well-being of female police officers. J Police Crim Psychol.

[104] Harris PA, Taylor R, Thielke R, Payne J, Gonzalez N, Conde JG (2009). Research electronic data capture (REDCap) - a metadata-driven methodology and workflow process for providing translational research informatics support. J Biomed Inform.

[105] Harris PA, Taylor R, Minor BL, Elliott V, Fernandez M, O’Neal L (2019). The REDCap consortium: building an International Community of Software Platform Partners. J Biomed Inform.

