# Supplementary material for: Mental health of working parents during the COVID-19 pandemic: can resilience buffer the impact of psychosocial work stress on depressive symptoms?
Source: BMC Public Health. 2022 Dec 26;22:2426. doi: 10.1186/s12889-022-14582-y (PMC9790816; doi:10.1186/s12889-022-14582-y)
Supplement: Supplementary file 1 — Additional file 1. Flowchart of participation rate and exclusion criteria resulting in final sample. The flowchart illustrates the stepwise applied exclusion criteria (no consent, respondance outside the given time frame, not having children yet, and no current employment, incomplete data). [file 12889_2022_14582_MOESM1_ESM.pdf]

Supplementary Figure 1

Flowchart of participation rate and exclusion criteria resulting in final sample

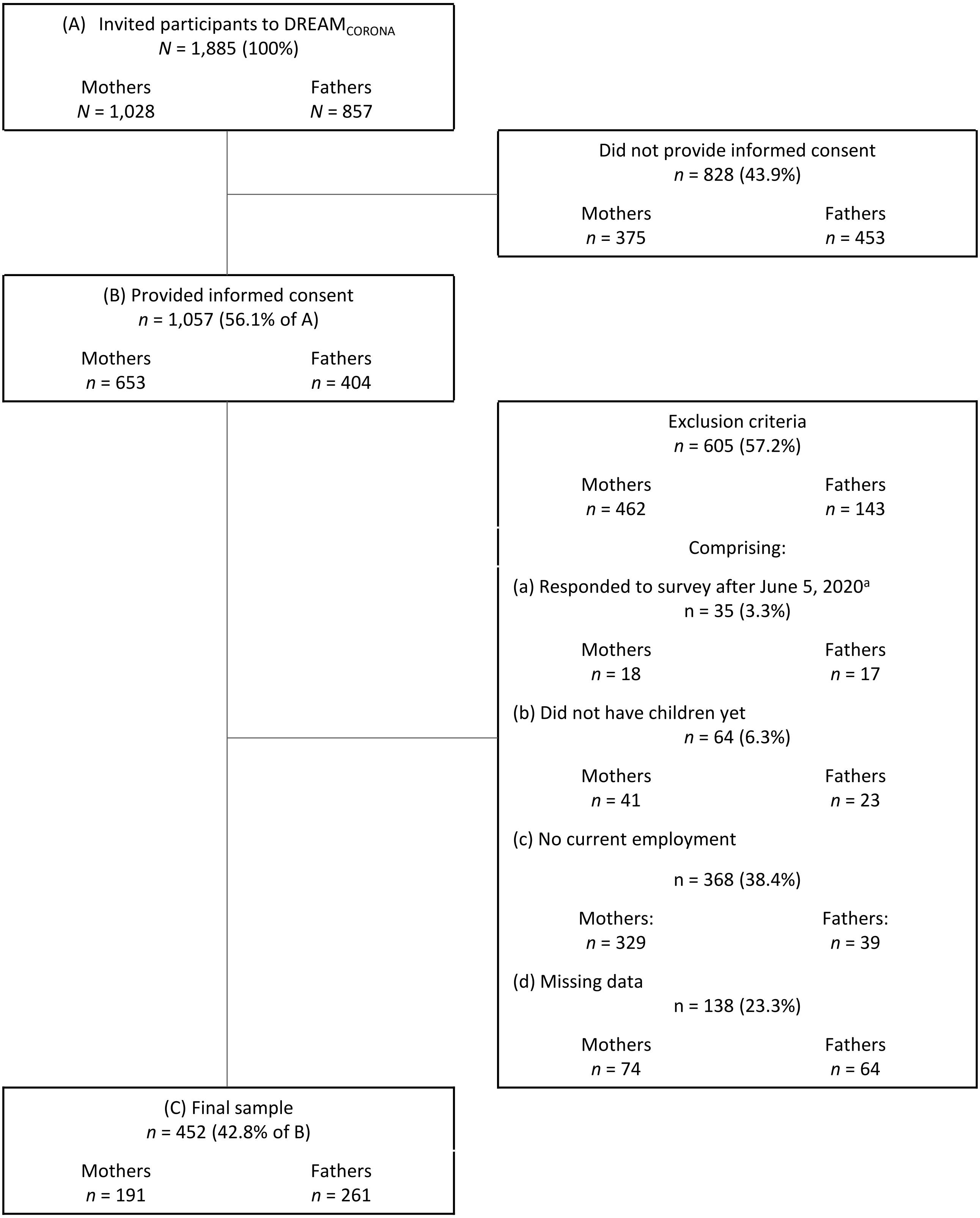

Note. Participation in the DREAM$_{CORONA}$ survey was possible from May 12 to October 1, 2020.

[a] To control for changes in political restrictions, participants who responded to the survey after June 5, 2020, were excluded as with June 6, 2020, new Covid-19 regulations came into effect
