# Supplementary material for: Mental health of working parents during the COVID-19 pandemic: can resilience buffer the impact of psychosocial work stress on depressive symptoms?
Source: BMC Public Health. 2022 Dec 26;22:2426. doi: 10.1186/s12889-022-14582-y (PMC9790816; doi:10.1186/s12889-022-14582-y)
Supplement: Supplementary file 2 — Additional file 2. Full results of multiple regression analysis. The included tables (Supplementary Table 1 and 2) show the full results of the multiple regression analysis of depressive symptoms on psychosocial work stress, resilience, and their interaction, while controlling for potential confounders. [file 12889_2022_14582_MOESM2_ESM.docx]

# **Full Results of Multiple Regression Analysis**

# **Supplementary Table 1** Full Results of Multiple Regression Analysis of Depressive Symptoms on WPC, Resilience, and Their Interaction, Controlled for Potential Confounders

|  | ***B*** | ***SE B*** | **β** | **95% CI** | | ***p*** | ***R²*** | ***F* for *ΔR²*** |
| --- | --- | --- | --- | --- | --- | --- | --- | --- |
|  |  |  |  | upper | lower |  |  |  |
| **Model 1** |  |  |  |  |  |  | .073 | 11.027^***^ |
| Constant | 7.356 | 0.676 |  | 5.907 | 8.781 | .000 |  |  |
| Gender | -1.634 | 0.461 | **-0.172** | -2.518 | -0.806 | .000 |  |  |
| Working hrs/w | -0.028 | 0.018 | -0.074 | -0.063 | 0.007 | .133 |  |  |
| LHD | 2.327 | 0.824 | **0.167** | 0.716 | 3.893 | .000 |  |  |
| **Model 2** |  |  |  |  |  |  | .205 | 69.022^***^ |
| Constant | 7.460 | 0.628 |  | 6.184 | 8.760 | .000 |  |  |
| Gender | -1.337 | 0.425 | **-0.141** | -2.124 | -0.582 | .002 |  |  |
| Working hrs/w | -0.035 | 0.017 | **-0.092** | -0.069 | -0.003 | .044 |  |  |
| LHD | 1.944 | 0.793 | **0.140** | 0.405 | 3.500 | .002 |  |  |
| WPC | 0.079 | 0.011 | **0.365** | 0.058 | 0.100 | .000 |  |  |
| **Model 3** |  |  |  |  |  |  | .348 | 91.540^***^ |
| Constant | 7.154 | 0.611 |  | 5.988 | 8.437 | .000 |  |  |
| Gender | -0.840 | 0.392 | **-0.088** | -1.539 | -0.161 | .034 |  |  |
| Working hrs/w | -0.030 | 0.016 | -0.079 | -0.063 | 0.003 | .057 |  |  |
| LHD | 0.813 | 0.684 | 0.059 | -0.478 | 2.159 | .151 |  |  |
| WPC | 0.072 | 0.010 | **0.334** | 0.052 | 0.091 | .000 |  |  |
| Resilience | -0.332 | 0.041 | **-0.394** | -0.407 | -0.244 | .000 |  |  |
| **Model 4** |  |  |  |  |  |  | .351 | 1.915 |
| Constant | 7.183 | 0.610 |  | 6.019 | 8.495 | .000 |  |  |
| Gender | -0.841 | 0.394 | **-0.089** | -1.551 | -0.180 | .034 |  |  |
| Working hrs/w | -0.030 | 0.016 | -0.079 | -0.062 | 0.003 | .056 |  |  |
| LHD | 0.804 | 0.684 | 0.058 | -0.506 | 2.156 | .155 |  |  |
| WPC | 0.073 | 0.010 | **0.336** | 0.053 | 0.092 | .000 |  |  |
| Resilience | -0.334 | 0.042 | **-0.397** | -0.412 | -0.246 | .000 |  |  |
| WPC x resilience | 0.002 | 0.002 | 0.055 | -0.001 | 0.006 | .167 |  |  |

*Notes*. *n* = 422. WPC = work-privacy conflict. hrs/w = hours per week. LHD = lifetime history of depression. Scores for WPC and resilience were mean-centered for regression analysis. Standard errors of *B* and 95% bias corrected and accelerated confidence intervals are based on 1,000 bootstrap samples. Δ*R²* ^*^ *p* < .05. ^**^ *p* < .01. ^***^ *p* < .001.

# **Supplementary Table 2** Full Results of Multiple Regression Analysis of Depressive Symptoms on ERI, Resilience, and Their Interaction, Controlled for Potential Confounders

|  | ***B*** | ***SE B*** | **β** | **95% CI** | | ***p*** | ***R²*** | ***F* for *ΔR²*** |
| --- | --- | --- | --- | --- | --- | --- | --- | --- |
|  |  |  |  | upper | lower |  |  |  |
| **Model 1** |  |  |  |  |  |  | .076 | 11.384^***^ |
| Constant | 7.455 | 0.696 |  | 5.995 | 8.926 | .000 |  |  |
| Gender | -1.649 | 0.469 | -0.173 | -2.555 | -0.725 | .000 |  |  |
| Working hrs/w | -0.032 | 0.019 | -0.082 | -0.069 | 0.006 | .096 |  |  |
| LHD | 2.339 | 0.832 | 0.168 | 0.579 | 3.987 | .000 |  |  |
| **Model 2** |  |  |  |  |  |  | .173 | 48.644^***^ |
| Constant | 7.849 | 0.655 |  | 6.517 | 9.096 | .000 |  |  |
| Gender | -1.670 | 0.443 | -0.175 | -2.524 | -0.776 | .000 |  |  |
| Working hrs/w | -0.042 | 0.018 | -0.109 | -0.076 | -0.010 | .019 |  |  |
| LHD | 2.076 | 0.797 | 0.149 | 0.413 | 3.675 | .001 |  |  |
| ERI ratio | 3.752 | 0.542 | 0.313 | 2.663 | 4.884 | .000 |  |  |
| **Model 3** |  |  |  |  |  |  | .298 | 47.713^***^ |
| Constant | 7.493 | 0.633 |  | 6.185 | 8.831 | .000 |  |  |
| Gender | -1.161 | 0.404 | -0.122 | -1.972 | -0.346 | .005 |  |  |
| Working hrs/w | -0.036 | 0.017 | -0.094 | -0.069 | -0.003 | .030 |  |  |
| LHD | 1.030 | 0.679 | 0.074 | -0.393 | 2.416 | .080 |  |  |
| ERI ratio | 2.748 | 0.531 | 0.230 | 1.685 | 3.815 | .000 |  |  |
| Resilience | -0.320 | 0.040 | -0.377 | -0.401 | -0.234 | .000 |  |  |
| **Model 4** |  |  |  |  |  |  | .308 | 5.668^*^ |
| Constant | 7.373 | 0.618 |  | 6.083 | 8.673 | .000 |  |  |
| Gender | **-1.118** | 0.400 | **-0.117** | -1.961 | -0.319 | .007 |  |  |
| Working hrs/w | **-0.036** | 0.017 | **-0.092** | -0.068 | -0.004 | .031 |  |  |
| LHD | 0.872 | 0.683 | 0.063 | -0.518 | 2.218 | .139 |  |  |
| ERI ratio | **3.041** | 0.552 | **0.254** | 1.918 | 4.104 | .000 |  |  |
| Resilience | **-0.315** | 0.039 | **-0.371** | -0.391 | -0.230 | .000 |  |  |
| WPC x resilience | -0.203 | 0.102 | **-0.101** | -0.418 | 0.005 | .018 |  |  |

*Notes.* *n* = 419. ERI = effort-reward imbalance. hrs/w = hours per week. LHD = lifetime history of depression. For regression analysis, the log-transformed value of the ERI ratio was used. Scores for ERI and resilience were mean-centered. Standard errors of *B* and 95% bias corrected and accelerated confidence intervals are based on 1,000 bootstrap samples. Δ*R²* ^*^ *p* < .05, ^**^ *p* < .01, ^***^ *p* < .001.
